# Young adult obese subjects with and without insulin resistance: what is the role of chronic inflammation and how to weigh it non-invasively?

**DOI:** 10.1186/1476-9255-6-6

**Published:** 2009-03-16

**Authors:** Giovanni Tarantino, Patrizia Colicchio, Paolo Conca, Carmine Finelli, Matteo Nicola Dario Di Minno, Marianna Tarantino, Domenico Capone, Fabrizio Pasanisi

**Affiliations:** 1 Department of Clinical and Experimental Medicine, Federico II University Medical School of Naples, Italy; 2 Department of Biomorphological and Functional Sciences, Federico II University Medical School of Naples, Italy; 3 Department of Neurosciences, Unit of Clinical Pharmacology, Federico II University Medical School of Naples, Italy

## Abstract

**Background:**

Obesity is a leading risk factor for metabolic syndrome whose further expression is non-alcoholic fatty liver disease. Metabolic syndrome is associated with a proinflammatory state that contributes to insulin resistance. Finally, a "metabolically benign obesity" that is not accompanied by insulin resistance has recently been postulated to exist.

**Aim:**

To find whether any inflammation markers were independently associated with the presence of insulin resistance, evaluating specific anthropometric, ultrasonographic and laboratory parameters in a population of young adult obese subjects.

**Methods:**

Of forty two young individuals, divided into two groups (with or without insulin resistance), were studied serum C-reactive protein and fibrinogen as indexes of chronic pro-inflammatory status. Body mass index, waist circumference and metabolic syndrome presence were assessed as part of the metabolic evaluation. Ultrasonography weighted visceral and subcutaneous abdominal fat thickness, spleen size as longitudinal diameter and liver hyperechogenicity.

**Results and Discussion:**

Serum C-reactive protein and fibrinogen as well as spleen longitudinal diameter were significantly increased in the obese young with insulin resistance compared to non-insulin resistance group. Insulin resistance was significantly associated with hepatic steatosis score at sonography (r = 0.33, P = 0.03), spleen longitudinal diameter (r = 0.35, P = 0.02) and C-reactive protein (r = 0.38, P = 0.01), but not with body mass index, visceral or subcutaneous abdominal adipose tissue, waist circumference and fibrinogen (P = 0.18, 0.46, 0.33, 0.37 and 0.4, respectively). Steatosis score at sonography was well associated with spleen volume (rho = 0.40, P = 0.01) and C-reactive protein levels (rho = 0.49, P = 0.002). Metabolic syndrome was much more frequent in obese patients with insulin resistance. These findings show that in young adults the only abdominal adiposity without insulin resistance, plays a scarce role in determining hepatic steatosis as well as metabolic syndrome.

**Conclusion:**

Increases in spleen size and CRP levels represent a reliable tool in diagnosing insulin resistance.

## Introduction

The risk of death from all causes enlarges throughout the range of moderate and severe increase of body weight for both men and women in all age groups [1].

Still, obesity is a leading risk factor for metabolic syndrome (MS) whose further expression is non-alcoholic fatty liver disease (NAFLD) [2], ranging from the "simple" fatty liver (FL) to the more severe form. i.e., non-alcoholic steatohepatitis (NASH), both of them characterized by progressive deposition of triglycerides in the hepatic parenchyma. Mechanisms involve low-grade inflammation in adipose tissue that contributes to insulin resistance (IR) [3]. The pattern of adipose tissue distribution, i.e., visceral adipose tissue (VAT) and subcutaneous abdominal adipose tissue (SAAT), is an important predictive factor of metabolic risk. Generally, visceral fat, better correlated with MS [4], is thought to be the most important factor for the development of hepatic steatosis in adult obese patients, but other Authors disagree [5].

Both SAAT and VAT can be easily and repeatedly measured by ultrasonography (US) for assessment of central obesity [6,7]. Furthermore, US is widely used to detect NAFLD, without exposing subjects to ionising radiations and with reduced costs. Obviously, a clear differentiation between FL and NASH in the spectrum of NAFLD is quite impossible without liver biopsy that is not always performed for ethical and technical issues. For this reason, we generally speak of hepatic steatosis (HS).

MS is associated with a prothrombotic and proinflammatory state. Among other biomarkers, C-reactive protein (CRP) and fibrinogen are strongly associated with the MS cluster, contributing in the MS risk assessment [8]. Although the relationship between inflammatory biomarkers and obesity/MS is not fully clear, central obesity with high visceral fat is strongly associated with blood level of CRP and IR in adults [9,10]. Another piece of research supports that for CRP and fibrinogen this relationship is determined by body adiposity and not by insulin sensitivity or glucose control [11]. On the other hand, NASH is characterized by increased spleen size and serum interleukin-6, both expression of low-grade chronic inflammatory status [12]. Furthermore, some Authorities have proposed CRP levels as reliable tool to help diagnose NASH and weigh NASH staging [13].

Finally, a "metabolically benign obesity" that is not accompanied by IR and early atherosclerosis has recently been postulated to exists in humans [14].

The aim of this study was to ascertain j) the differences, if any, between young obese individuals with such an apparently harmless phenotype without IR and young obese individuals with IR, jj) the correlation between the entity of VAT and SAAT deposition and the severity of HS, all of them studied by US in basal condition, jjj) to find among the inflammation markers, comprehending spleen size, which one was independently associated with the presence of IR and, consequently, HS.

## Methods

We carried out a cross-sectional study at an University Medical Center trying to clarify some correlations between IR and NAFLD, liver disease almost characteristic of young obese patients [15].

Protocol was consistent with the principles of the Declaration of Helsinki and participants gave their informed consent.

### Population

Forty two young obese individuals were selected to participate in this study before being given weight control indications, forming two groups well-matched for age, body mass index (BMI), waist circumference (WC) and sex. They had no family history of diabetes and showed a normal serum glucose level at the enrollment time. The subjects were classified as insulin sensitive or insulin resistant according to a stringent homeostasis model assessment of IR (HOMA) cut-off (>or = 1.95 as insulin resistant) [16]. Exclusion criteria were a history of infectious chronic diseases including hepatitis B and C, neoplastic and/or haematological diseases, autoimmune and storage diseases, unstable medical conditions, drugs inducing hepatic steatosis and prior use of medications known to affect inflammation, blood lipids or insulin sensibility and finally the practice of playing sport. Alcohol abuse was ruled out, according to the DSM-IV diagnostic criteria, by means of screening tests such as MAST (Michigan Alcohol Screening Test) and CAGE (Cut down, Annoyed, Guilty, and Eye opener), as well as random tests for blood alcohol concentration and the use of a surrogate marker, e.g., Mean Corpuscular Volume.

They were fully investigated for the presence of MS stigmata and NAFLD. Subjects were consecutively enrolled when having second or third degree obesity on the basis of BMI cut-off points of >34.9 kg/m^2 ^and >39.9 kg/m^2^, respectively. Central obesity was identified by waist circumference WC >102 cm in men or >88 cm in women, measured at the midpoint between the lower border of the rib cage and the iliac crest. MS was defined according to the revised Adults Treatment Panel III (2001), and three or more criteria were considered: plasma glucose concentration of at least 100 mg dL-^1^, WC >102 cm in men and >88 cm in women, serum high-density lipoprotein (HDL)-cholesterol concentration <40 mg dL^-1 ^in men and <50 mg dL^-1 ^in women, blood pressure of at least 130/85 mm Hg, and serum triglyceride concentration of at least 150 mg dL^-1^. NAFLD was associated with recent US features of "bright liver", with or without aminotransferase increase of unknown origin.

Sonographic measurements were performed using an ESAOTE *Technos *(Genoa, Italy). Briefly, transverse scanning was performed to measure the SAAT using a 7.5 MHz probe and VAT using a 3.5 MHz probe, 1 cm above the umbilicus. The SAAT was defined as the thickness between the skin-fat interface and the linea alba, and the VAT was defined as the distance between the anterior wall of the aorta and the internal face of the recto-abdominal muscle perpendicular to the aorta. When the aortic walls were not visualized as they were obscured by bowel gas, the doppler scan was used.

Spleen longitudinal diameter (SLD) was performed by postero-lateral scanning. The maximum length (the optically greatest overall longitudinal dimension obtained from one of the two poles) and the cranio-caudal length (the optically maximal transversal dimension intercepting one of the two poles) were measured; the resulting values were then averaged, since the two measurements do not always coincide.

All the indices were measured thrice directly from the frozen images using an electronic caliper.

The classification of "bright liver" or HS was based on the following scale of hyperechogenity: 0 = absent, 1 = light, 2 = moderate, 3 = severe, pointing out the difference between the densities of the liver and the right kidney.

The intra-inter-observational reproducibility of the sonographic estimations was 1.9, 4.1, 2.5, 2.3% and 1.7, 4.8, 2.6, 2.4% for the bright liver, VAT, SAAT and the SLD respectively, not so far from previous observations, [17]. HOMA was assessed by the formula: fasting insulin (μU/mL) × fasting glucose (mg/dL)/405 [18].

### Analytes

Serum concentrations of alanine aminotransferase (ALT) were performed by in-house procedures with normal values set down below 35 U/L. High sensitivity serum CRP was dosed by an enzyme immunoassay kit of BioCheck Inc, Foster City CA, USA, with lower limit of 0.1 mg/L and CVs of intra- and inter-assay precision of 2.3–7.5 and 2.5–4, respectively. To quantify fibrinogen in serum was used the Human Fibrinogen, ELISA Kit by GenWay Biotech Inc, San Diego CA, USA, with normal values placed between 200 and 400 mg/L. Serum-free insulin concentrations were determined by double antibody radioimmunoassay (Pharmacia Insulin RIA Kit by Pharmacia Uppsala, Sweden). The fasting levels for healthy individuals lay between 3 and 15 μU/mL

### Statistics

Variables were expressed as mean +/- SD or median with 25–75 percentiles and according to the distribution of same ones (uniformly or not uniformly, respectively), it was used Student t test or Mann-Witney U test. Correlation in the whole population was assessed by Pearson's r or Spearman's rho as per the type of variable. Chi square studied frequencies. Logistic regression, using as independent variables CRP, ALT, SAAT, VAT, SLD and HS at US, was evaluated to predict IR.

## Results

In order to allow readers to gauge how well the study findings apply to their patients (external validity) we stress that forty two subjects divided into two groups of 21 each one, well balanced for gender (chi square 1.5, P = 0.2), BMI (P = 0.25), WC (P = 0.45) and age (P = 0.79), but different for HOMA (P = < 0.001) were studied in a cross-sectional fashion, Table 1. In Table 2 are evidenced the hepatic damage (P = 0.87), SAAT (P = 0.42), VAT(P = 0.26), and SLD at US, the only one significantly different. The behavior of serum CRP, fibrinogen and HS at US, all statistically significant, is showed in Table 3.

**Table 1:** Characteristics of studied population

	**IR Absent**		**IR Present**	
	Mean	SD	Mean	SD
AGE (years)	19.5	2.4	19.2	3.3
HOMA**∞**	1.4	0.4	4.0	1.7
BMI	39.4	5.5	41.4	6.1
WC cm	118.9	13.0	121.7	10.5

Body mass index (BMI); homeostasis model assessment of insulin resistance (HOMA); waist circumference (WC); insulin resistance (IR); **∞ **P < 0.001; standard deviation (SD).

**Table 2:** Ultrasonography and laboratory data

	**IR Absent**		**IR Present**	
	Mean	SD	Mean	SD
VAT (mm)	55.2	16.1	61.4	18.0
SAAT (mm)	41.1	5.8	39.7	5.2
SLD● (mm)	113.8	13.1	122.1	8.9
ALT (U/L)	26.2	16.5	25.6	6.1

Visceral adipose tissue (VAT); subcutaneous abdominal adipose tissue (SAAT); spleen longitudinal diameter (SLD); alanine aminotransferase (ALT); insulin resistance (IR); [black circle] P = 0.02; standard deviation (SD).

**Table 3:** Further ultrasonography and laboratory data

	**IR Absent**		**IR Present**	
	Median	25–75 P	Median	25–75 P
CRP **∞ **mg/L	0.31	0.3–0.5	1.47	0.8–2.22
Fibrinogen● mg/L	345.0	306.7–382	411.0	363.7–466.2
HS score**† **at US	0	0–1	2	0.7–2

Insulin resistance (IR); C-reactive protein (CRP); severity of hepatic steatosis at ultrasonography (HS score at US); [black circle] P = 0.04; **∞ **P < 0.001; [black cross] P = 0.003; Percentile (P).

HOMA was significantly associated with HS score at US (r = 0.33, P = 0.03), SLD (r = 0.35, P = 0.02) and CRP (r = 0.38, P = 0.01), but not with BMI, SAAT, VAT, WC and fibrinogen (P = 0. 26, 0.46, 0.33, 0.37 and 0.4, respectively). CRP serum concentrations well correlated to spleen volume measured as SLD at US (r = 0.32, P = 0.04). ALT activity was significantly associated with BMI values (r = 0.33, P = 0.04) and mostly with HS score at US (rho = 0.44, P = 0.004). SLD displayed a non significant correlation to WC (r = 0.29, P = 0.06) and BMI (r = 0.18, P = 0.3). At US, HS was associated with SLD (rho = 0.40, P = 0.01). The strongest association was found when comparing CRP levels with HS score at US (rho = 0.49, P = 0.002). MS was much more frequent in IR group (chi square 9.6, P = 0.002). Only CRP strongly predicted the presence of IR (coefficient 11.5 ± 5.2, P = 0.03).

## Discussion

Authorities give emphasis on various facts: j) the prevalence of obesity is increasing worldwide, especially in the young adult population; jj) obesity is characterized by a state of low-grade chronic inflammation at all ages; jjj) although VAT is more highly correlated with metabolic risk factors even after accounting for standard anthropometric indexes, it is possible that SAAT actually contributes a more absolute risk because SAAT volume is greater than VAT [19]; jjjj) in adults, visceral fat is directly associated with liver inflammation and fibrosis independently of insulin resistance and HS and it should be a central target for future interventions in NAFLD and indeed in all the metabolic diseases [20]. On the other hand, a recent observation has highlighted the role of spleen as further non invasive marker of chronic low-grade inflammation in "dysmetabolic" patients with obesity and/or visceral adiposity [12].

Commenting on the results, we can observe that our data in young obese patients do not support an association between anthropometric measurements and HOMA. HOMA was well correlated to severity of HS and to the spleen size, both evaluated at US. The HS score was different in the two groups, so the MS detection. The main inflammatory index (CRP) and SLD behaved like HOMA, suggesting a strict link between inflammatory status and IR, but not necessarily in this sequence. In other words, enhanced spleen size, HS presence and CRP levels represented a reliable tool in confirming IR. These findings add to the body of pertinent knowledge the concept that a partially "benign" obesity, characterized by absence of IR, lack of HS or at the most presence of "light" form, lower or normal serum levels of inflammatory markers and, in few cases, minimal elevation of aminotransferase can exist in this particular population, at least at the moment of study.

Discussing possible mechanisms and explanations for our findings, we can argue that IR presence is the most important factor in determining the fat deposition in organs (in our case liver) unrelated to BMI, visceral and subcutaneous abdominal adipose tissue stores. If IR has a genetic determinant or is a phenotypic expression, it remains to be established. Although it is generally thought that organ deposition starts occurring when visceral and subcutaneous abdominal adipose tissue stores are full, we were not able to confirm this point. Being IR not related to the entity of fat deposition, we hypothesize that the chain of events does not presuppose the obesity as if it was the cause of IR; this fact is supported by the clear association between the inflammatory status (CPR levels and spleen volume) and the HS score. Could the high fat liver content be the breaking point between "benign" and "progressive" obesity? This is the first intriguing question that could be answered in the course of successive follow-ups of this population, as work in progress. Comparing study results with relevant findings from other published work, we found a possible confirmation in a study that suggests that the contribution of visceral fat to inflammation may not be completely accounted for by clinical measures of obesity (BMI and WC, [21]). A second point of the problem to stress is whether the weight control can slow down the progression of IR and the worsening of fat deposition in organs in these obese young patients as for overweight subjects [22].

Furthermore, although insulin sensitivity was negatively correlated with liver fat content in overweight and moderately obese pre-pubertal children, inflammation markers were not correlated with insulin sensitivity [23], contrary to adults. This last aspect makes gain ground to the hypothesis that IR presence is associated with inflammation after many years and probably liver produces great amount of CRP to reacts to its continuous fat deposition. Having found in "dysmetabolic" patients a spleen volume increased strengthens this hypothesis and proposes this measurement as a non-invasive parameter to weigh the grass-roots low-grade chronic inflammatory process.

Discussing the limitations of the present study we have to pinpoint that liver biopsy is currently used to distinguish between 'simple' fatty liver and NASH, or stage the degree of fibrosis accurately, even though a recent study has demonstrated a good correlation between liver histology and US features [24]. It is very likely that US will be very often used in clinical practice for the routine assessment of regional adiposity [25], although other methods are more specific; among these, magnetic resonance imaging is fairly well established [26], together with magnetic resonance spectroscopy [27]. US imaging is also emerging as useful method for quantification of VAT, SAAT and tissue fat content in vivo for therapeutic interventions, i.e., when repeated measures are requested after diet programs. Any crucial future research directions should consider the reliability of this imaging tool.

The clinical implications summarized in a straightforward and circumspect manner of the work is that we believe that our data reinforce the need to start weight control at the "earliest possible time" in the progression of disease, i.e., obesity/MS, which means diagnosing NAFLD earlier, rather than later by the means of the simplest method possible, i.e., at US.

## Abbreviations

(BMI): Body mass index; (HOMA): homeostasis model assessment of insulin resistance; (NAFLD): non-alcoholic fatty liver disease; (NASH): non-alcoholic steatohepatitis; (HS): hepatic steatosis; (IR): insulin resistance; (MS): metabolic syndrome; (VAT): visceral adipose tissue; (SAAT): subcutaneous abdominal adipose tissue; (CRP): C-reactive protein; (WC): waist circumference; (SLD): spleen longitudinal diameter; (US): ultrasonography; (ALT): alanine aminotransferase; (SD): standard deviation.

## Competing interests

The authors declare that they have no competing interests.

## Authors' contributions

CP conceived of the study, TG drafted the manuscript. FC, MND, CD and PF helped to draft the manuscript. TM and TG performed ultrasound. TG participated in the design of the study and performed the statistical analysis. All authors read and approved the final manuscript.
